# Oyster-Derived Zinc-Binding Peptide Modified by Plastein Reaction via Zinc Chelation Promotes the Intestinal Absorption of Zinc

**DOI:** 10.3390/md17060341

**Published:** 2019-06-08

**Authors:** Jianpeng Li, Chen Gong, Zaiyang Wang, Ruichang Gao, Jiaoyan Ren, Xiaodong Zhou, Haiyan Wang, He Xu, Feng Xiao, Yuhui Cao, Yuanhui Zhao

**Affiliations:** 1 College of Food Science and Engineering, Ocean University of China, Qingdao 266003, China; ljp19881988@126.com (J.L.); 17664076298@163.com (C.G.); wzy13092440827@163.com (Z.W.); caoyuhuijy@126.com (Y.C.); 2 School of Food and Bioengineering, Jiangsu University, Zhenjiang 212013, China; xiyuan2008@ujs.edu.cn; 3 School of Food Sciences and Engineering, South China University of Technology, Guangzhou 510641, China; renjiaoyanscut@126.com; 4 Hisense (Shandong) Refrigerator Co., Ltd., Qingdao 266100, China; zhouxiaodong@hisense.com (X.Z.); wanghaiyan8@hisense.com (H.W.); 5 Jiangsu Baoyuan Biotechnology Co., Ltd., Lianyungang 222100, China; xuhe-2001@sohu.com; 6 College of Food and Bioengineering, Henan University of Science and Technology, Luoyang 471023, China; xfeng@haust.edu.cn

**Keywords:** oyster zinc-binding peptide, peptide-zinc complex, caco-2 cells, intestinal absorption, zinc bioavailability

## Abstract

Zinc-binding peptides from oyster (*Crassostrea gigas*) have potential effects on zinc supplementation. The aim of this study was to prepare efficient zinc-binding peptides from oyster-modified hydrolysates by adding exogenous glutamate according to the plastein reaction and to further explore the zinc absorption mechanism of the peptide-zinc complex (MZ). The optimum conditions for the plastein reaction were as follows: pH 5.0, 40 °C, substrate concentration of 40%, pepsin dosage of 500 U/g, reaction time of 3 h and l-[1-^13^C]glutamate concentration of 10 mg/mL. The results of ^13^C isotope labelling suggested that the addition of l-[1-^13^C]glutamate contributed to the increase in the zinc-binding capacity of the peptide. The hydrophobic interaction was the main mechanism of action of the plastein reaction. Ultraviolet spectra and scanning electronic microscopy (SEM) revealed that the zinc-binding peptide could bind with zinc and form MZ. Furthermore, MZ could significantly enhance zinc bioavailability in the presence of phytic acid, compared to the commonly used ZnSO_4_. Additionally, MZ significantly promoted the intestinal absorption of zinc mainly through two pathways, the zinc ion channel and the small peptide transport pathway. Our work attempted to increase the understanding of the zinc absorption mechanism of MZ and to support the potential application of MZ as a supplementary medicine.

## 1. Introduction

Zinc, as an essential micronutrient, is essential for human health and participates in numerous enzymatic and metabolic processes in human organisms [1]. The human body mass contains 2–3 g of zinc [2]. In the human body, zinc deficiency usually leads to serious consequences, such as growth defects, hypogonadism, and neurological dysfunctions [3]. As of 2012, zinc deficiency is estimated to affect up to two billion people worldwide [4], which represents a significant global burden. Thus, it is necessary to improve zinc bioavailability and absorption for optimum health. The fortification of food by adding zinc salts or zinc-chelating peptides has promise as an intervention strategy. However, the most commonly used ZnSO_4_ could irritate the gastrointestinal mucosa and is not safe for long-term intake [5]. In addition, the efficacies of mineral supplements are strongly blocked by mineral absorption inhibitors (such as phytic acid) in daily diets [6]. Thus, searching for both effective and safe components from seafood sources as alternatives to prevent zinc deficiency is a research area with urgent need worldwide.

Oyster (*Crassostrea gigas*), a member of the ostreidae family [7], is a good source of proteins, essential fatty acids, and other nutrients [8]. Many studies have shown that oyster, mainly oyster peptides, has remarkable pharmacological efficacies, including antimicrobial, antihypertensive, anticancer, antioxidant, and antiviral abilities [9,10,11,12]. Oysters are usually richer in zinc elements than other seafoods, and zinc is easily combined with proteins or amino acids in oyster soft tissue [13]. The zinc-chelating peptides extracted from oyster have attracted wide attention. Work by Chen et al. demonstrated that the peptide (HLRQEEKEEVTVGSLK) produced from oyster protein hydrolysis has a marked ability to bind zinc [14], and Zhang et al. revealed that the hydrolysate-zinc complex (OPH-Zn) can improve zinc bioaccessibility [15]. However, the zinc absorption mechanism of the oyster-derived peptide-zinc complex has not been fully studied. Thus, exploring the effect of the peptide-zinc complex on zinc supplementation and improving our understanding of the mechanism involved may advance the understanding of oyster peptides as zinc supplementation agents.

Recently, it has been reported that zinc-binding peptides can be obtained from oyster protein hydrolysates by a facile plastein reaction [16]. The plastein reaction was first discovered by Danilevski in 1902 when he added chymotrypsin to protein hydrolysates, and this reaction is considered to be a reverse enzymatic reaction [17]. Under normal conditions, bioactive peptides remain inactive in the sequence of their parent proteins. However, with appropriate enzymatic hydrolysis, the peptides can be released and activated. The plastein reactions provided a possible method to synthesize a multifunctional peptide-based ingredient with desirable bioavailability, safety, and functional properties [18]. However, due to the complexity of the reaction, the mechanism of plastein reactions remains unclear and is still an intellectual curiosity.

In the present study, the efficient zinc-binding peptides were prepared from oyster-modified hydrolysates according to the plastein reaction and the mechanism of the plastein reaction was explored. Then the peptide-zinc complex (MZ) was prepared and characterized. The zinc bioavailability of MZ in the presence of phytic acid was investigated in Caco-2 cells. Subsequently, a possible zinc absorption mechanism of MZ in Caco-2 cells was further explored. This work could provide new information for the potential application of oyster protein-based zinc supplements.

## 2. Results and Discussion

### 2.1. Optimization of the Plastein Reaction Conditions

The synthetic reaction dominated the plastein reaction and it inevitably led to a decrease in the free amino acids in the reaction system [17,19]. To improve the efficiency of the plastein reaction, the effects of hydrolysis pH, temperature, substrate concentration, pepsin dosage, reaction time, and glutamate concentration on the decrease in the free amino acid content of the plastein products were optimized in the present study.

**Figure 1:**
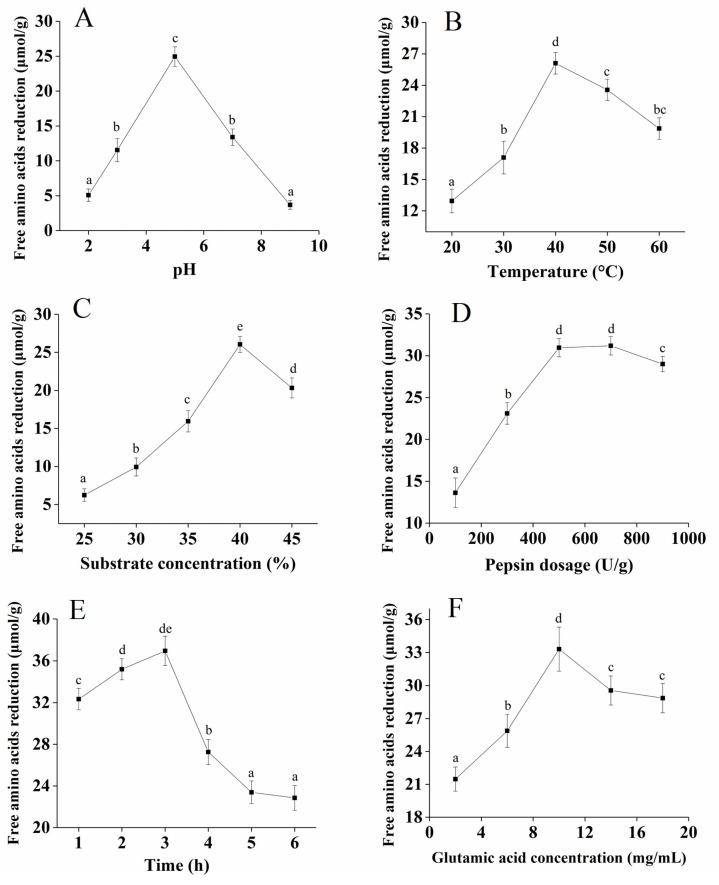
Effects of hydrolysis pH (**A**), temperature (**B**), substrate concentration (**C**), pepsin dosage (**D**), reaction time (**E**), and glutamate concentration (**F**) on free amino acids reduction of the plastein products. Unless otherwise noted, temperate was 40 °C, pH was 5.0, substrate concentration was 40%, pepsin dosage was 500 U/g, reaction time was 3 h, and l-[1-^13^C]glutamate concentration was 10 mg/mL. Each point is shown as the means ± standard deviation (SD) (*n* = 3). Different letters indicate significant differences (*p* < 0.05).

The effect of pH on free amino acid reduction was significant, as shown in Figure 1A. With an increase in pH, the reduction of free amino acid presented an upward trend, reaching a maximum value at pH 5.0, and then presented a downward trend at a higher pH. pH can affect the binding efficiency of enzyme molecules and substrates [20]. The suitable pH of pepsin used in this study ranged from 1.5 to 5.5 [21]. Therefore, the optimal hydrolysis pH was 5.0. The free amino acid reduction gradually increased from 20 to 40 °C, and the maximum value was reached at 40 °C (Figure 1B). The possible reason for this finding is that pepsin was gradually activated as the temperature of the enzymatic hydrolysis increased, and the structure of the enzyme protein was affected when the temperature exceeded the optimum temperature. Therefore, the optimal temperature was determined to be 40 °C. Figure 1C shows that when the substrate concentration increased from 25% to 40%, the free amino acid reduction gradually increased, reaching a maximum value at 25 µmol/g. Therefore, the optimal substrate concentration was determined to be 40%. Figure 1D shows that when the pepsin dosage increased from 100 U/g to 500 U/g, the free amino acid reduction gradually increased, which probably meant that the enzyme binding sites were completely occupied by the substrate. With an increase in the reaction time, the free amino acid reduction increased to a maximum value at 3 h (Figure 1E). Therefore, 3 h was selected as the appropriate holding time. The effect of the glutamate concentration on the free amino acid reduction was significant, as shown in Figure 1F, and the optimal glutamate concentration was determined to be 10 mg/mL. The addition of exogenous glutamate during the plastein reaction can effectively increase the efficiency of the plastein reaction.

Overall, the optimum conditions for the free amino acid reduction of the plastein products were obtained at: pH 5.0, 40 °C, substrate concentration of 40%, pepsin dosage of 500 U/g, reaction time of 3 h, and glutamate concentration of 10 mg/mL.

### 2.2. Change in Zinc-Binding Capacity and Hydrophobicity during the Plastein Reaction

**Figure 2:**
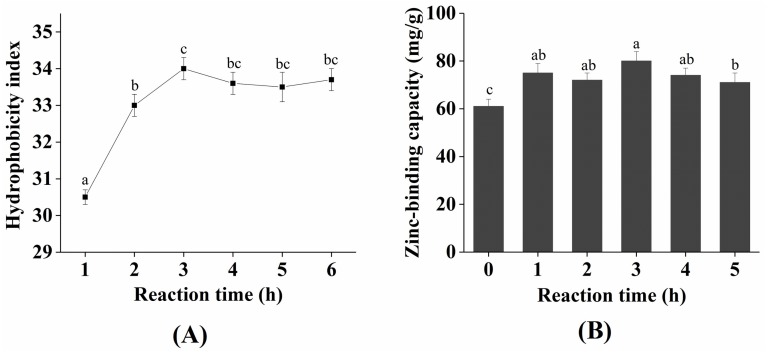
Change in hydrophobicity (**A**) and zinc-binding capacity (**B**) during the plastein reaction. Each point is shown as the means ± SD (*n* = 3). Different letters indicate significant differences (*p* < 0.05).

As shown in Figure 2A, the hydrophobicity index of the plastein products steadily increased over 3 h, attaining a maximum of 33.9%. Our results are consistent with those of Jiang et al. [19], who reported that the internal hydrophobic amino acids were exposed during the plastein reaction, indicating that hydrophobic interactions are important forces in the formation of plastein products. Similarly, the zinc-binding capacity of the plastein products continuously increased for 3 h (Figure 2B). This result is consistent with that of Chen et al. [14]. The possible reason for this finding was that the hydrophilic groups (−OH, −NH_2_, −COOH) were exposed during the reaction, which provided additional binding sites for the zinc ions. In addition, the added exogenous glutamate increased the −COOH content and thus led to an increase in the binding capacity of zinc ions.

### 2.3. The Effects of Protein Denaturants on the Stability of Plastein Products

**Figure 3:**
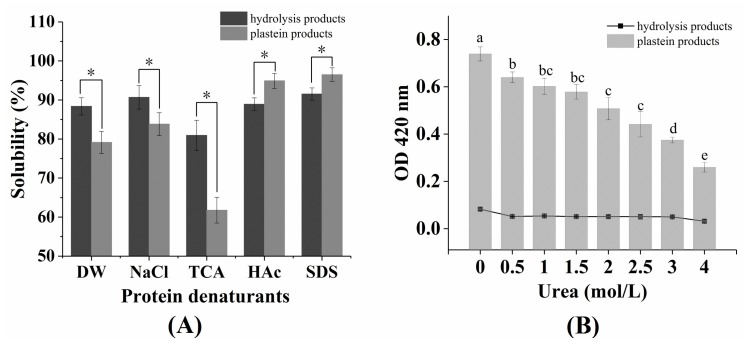
(**A**) Solubility of plastein products in different denaturants; (**B**) effect of urea on the stability of plastein products. Abbreviations: DW, deionized water; NaCl, sodium chloride; TCA, trichloroacetic acid; HAc, acetic acid; SDS, sodium dodecyl sulfate. Each point is shown as the means ± SD (*n* = 3). Asterisk (*) and different letters indicate significant differences (*p* < 0.05).

As shown in Figure 3A, the solubility of the plastein products in the deionized water (DW) and sodium chloride (NaCl) groups was significantly lower than in the hydrolysis products (*p* < 0.05), suggesting that the hydrophobicity of the plastein products was high. Sodium dodecyl sulfate (SDS) and acetic acid (HAc) can destroy the protein structures that are maintained by hydrophobic interactions and then dissolve the plastein products [22]. The solubility of the plastein products in the HAc and SDS groups was significantly higher than the solubility of the hydrolysis products (*p* < 0.05) (Figure 3A), suggesting that the hydrophobic interactions may be primarily responsible for the formation of plastein products, which was consistent with the conclusion of Figure 2A. In addition, high molecular weight proteins have low solubility in trichloroacetic acid (TCA) [23]. The solubility of the plastein products in the TCA group was significantly lower than that of the hydrolysis products (*p* < 0.05) (Figure 3A), suggesting that the plastein products had a higher molecular weight than the hydrolysis products. Urea is a polar molecule that can destroy hydrogen bonds in the protein [24]. As shown in Figure 3B, urea had a significant effect on the turbidity value of the plastein products (*p* < 0.05), which suggested that hydrogen bonds may be partially responsible for the formation of plastein products.

### 2.4. Change in Molecular Weight Distribution during the Plastein Reaction

**Figure 4:**
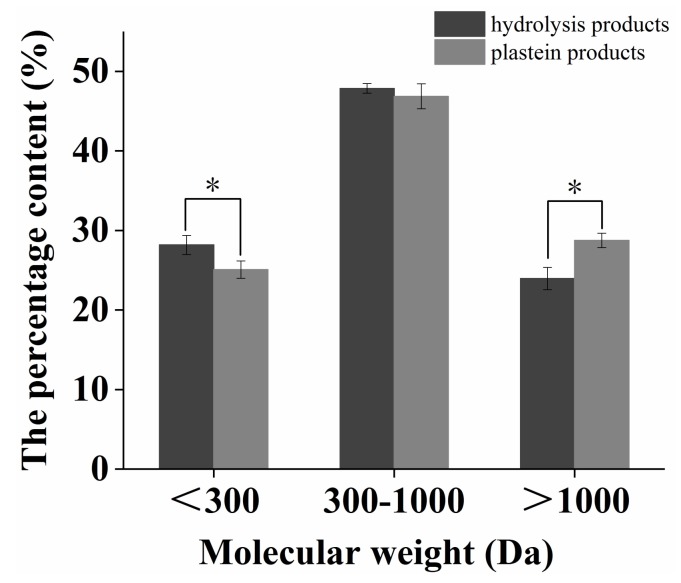
The change of molecular weight distribution during plastein reaction. Each point is shown as the means ± SD (*n* = 3). Asterisk (*) indicate significant differences (*p* < 0.05).

As shown in Figure 4, after the plastein reaction, the content of plastein products with a molecular weight greater than 1000 Da significantly increased, while the content of plastein products with a molecular weight less than 300 Da significantly decreased (*p* < 0.05), indicating that the small molecular weight glutamate and polypeptide were bound to other polypeptide chains by transpeptidation and condensation reactions, thus increasing the percentage of macromolecular polypeptides.

Combined with the above experimental results, it could be demonstrated that the hydrophobic interaction was the main mechanism of action of the plastein reaction and there was also a relatively weak condensation and transpeptidation reaction.

### 2.5. Zinc-Binding Capacity and l-[1-^13^C]Glutamate Abundance of Different Components of Plastein Products

**Figure 5:**
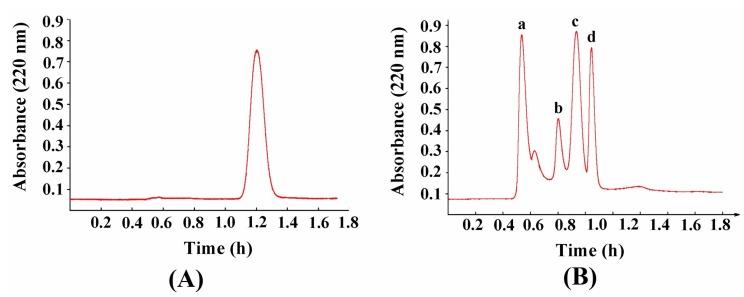
Sephadex G-15 chromatograph of phenylalanine standard (**A**) and different components of plastein products (**B**), a–d mean the fractions that obtained at 220 nm.

The conjugated double bond in the peptides and phenylalanine has an ultraviolet characteristic absorption peak at 220 nm. An aqueous solution of phenylalanine showed an absorption peak between 1.1 and 1.4 h (Figure 5A), indicating that most of the free amino acids eluted with the mobile phase mainly after 1.1 h. The fractions a–d were obtained at 220 nm (Figure 5B). Afterwards, these components were collected to determine the zinc-binding capacity and l-[1-^13^C]glutamate abundance. The higher the l-[1-^13^C]glutamate abundance was, the higher the ratio of ^13^C/^12^C in the sample. The zinc-binding capacity of a and b components was significantly higher than that of c and d components, meanwhile the l-[1-^13^C]glutamate abundance of a and b was also significantly higher than that of c and d (*p* < 0.05) (Table 1), suggesting that the addition of exogenous l-[1-^13^C]glutamate contributed to an increase in the zinc-binding capacity of the peptides.

In addition, the zinc-binding capacity of the d component was significantly lower than that of the b component (*p* < 0.05), which may be related to the molecular weight and spatial structure of the polypeptides. Some macromolecular peptides were aggregated into hydrophobic macromolecular peptides by hydrophobic interactions during the plastein reaction, thereby resulting in the change of structure of the polypeptides and the reduction of binding sites for the zinc ions.

### 2.6. Scanning electron microscopy (SEM) Photograph and UV-Vis Absorption of the peptide-zinc complex (MZ)

**Figure 6:**
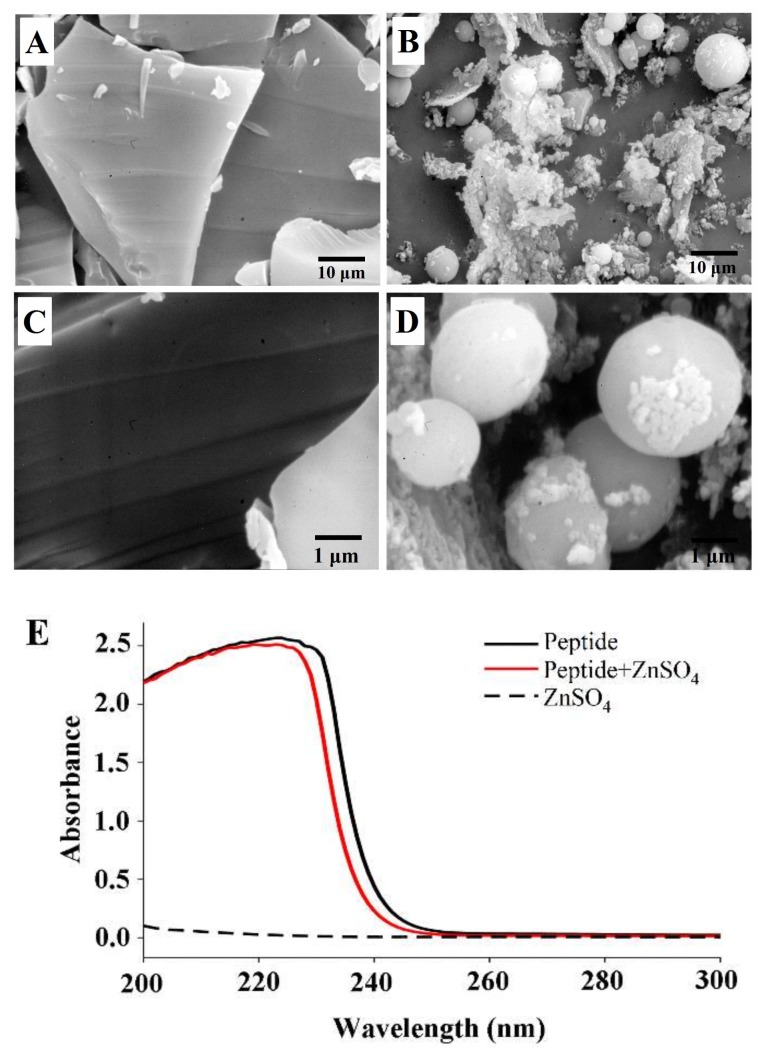
Scanning electron microscopy (SEM) photograph of peptide (**A**,**C**) and peptide-zinc complex (MZ) (**B**,**D**), UV-vis absorption analysis (**E**).

In our previous study, the peptide Glu-Val-Pro-Pro-Glu-Glu-His (M) with high zinc-binding capacity was isolated and purified [16], and we further prepared the MZ in this study. The peptide M showed a sheet appearance with a smooth surface (Figure 6A,C). In contrast, MZ demonstrated a granule-like structure, which was markedly different from the peptide M image (Figure 6B,D). Metal ions can interact with peptides and facilitate peptide aggregation [25]. Zhang et al. [15] reported that zinc ions can promote aggregation by increasing the speed of the dimerization of the peptides. In addition, the carbonyl oxygen and hydroxyl oxygen on the carboxyl group in the peptide chain are involved in the coordination of zinc ions [16], thus promoting the formation of aggregates. The SEM results suggest that the interaction between the peptide and zinc ions could lead to the formation of particles.

The peptide M had a maximum absorption peak at 223 nm, while the mixture of zinc-binding peptide and ZnSO_4_ had the maximum absorption peak shifted to 220 nm (Figure 6E). Previous studies have indicated that the addition of zinc ions affected the spatial structure of the peptide [14], thus inducing hypochromicity in the UV absorption spectrum. From these results, it could be concluded that the peptide M could bind with zinc and form the MZ.

### 2.7. Cytotoxicity of MZ Against Caco-2 Cells

**Figure 7:**
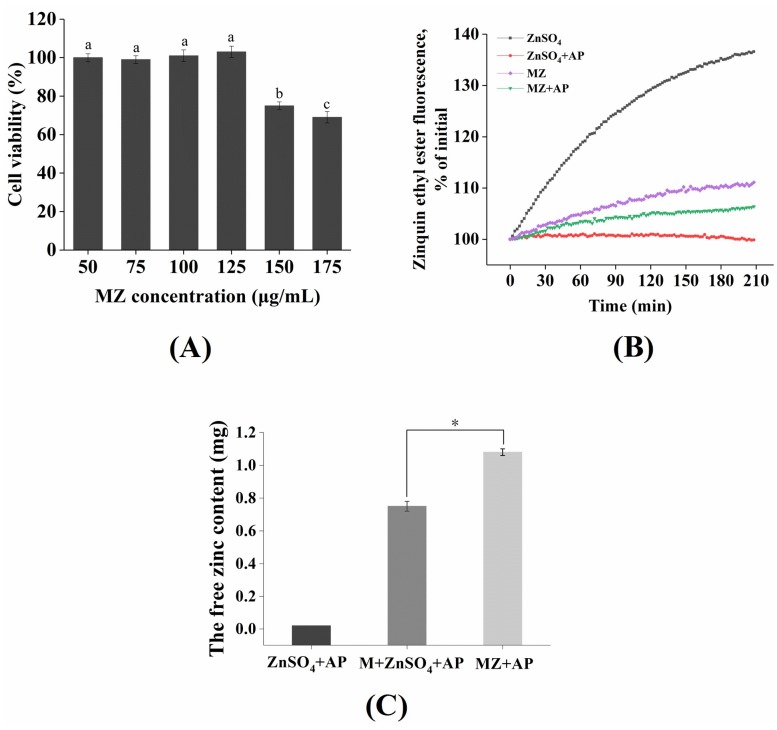
(**A**) Cell viability of Caco-2 cells after being incubated with the MZ at different concentrations. (**B**) Zinc uptake by Caco-2 cell monolayers in the presence of 2:1 molar ratio of zinc/phytic acid. Cellular uptake of exogenous zinc was measured as the quenching of Zinquin ethyl ester fluorescence. (**C**) The free zinc content after phytic acid treatment. M: peptide (EVPPEEH); MZ: peptide-zinc complex; AP: phytic acid. Different lowercase letters and asterisk (*) indicates significant difference between groups (*p* < 0.05).

The MZ exhibited no significant cytotoxicity in Caco-2 cells at a concentration of 125 µg/mL or less, however, MZ significantly reduced the viability of Caco-2 cells at concentrations over 125 µg/mL (*p* < 0.05) (Figure 7A). Therefore, subsequent absorption and transport experiments were conducted at a dose of 125 µg/mL.

### 2.8. Absorption of Zinc from MZ in Caco-2 Cells

Caco-2 cells are similar to intestinal epithelial cells in structure and function, so they are commonly used to simulate the absorption characteristics of intestinal epithelial cells in vitro [26]. Usually, exogenous zinc enters the cellular zinc pool [27]. *N,N,N’,N’*-tetrakis (2-pyridylmethyl)-ethylene-diamine (TPEN), a zinc ion chelator, has frequently been used to consume the initial free zinc in cells [28], and then the Zinquin ethyl ester fluorescent probe is usually used to monitor cellular zinc uptake in real time [29]. As shown in Figure 7B, zinc uptake from ZnSO_4_ and MZ, at concentrations equivalent to 96 μM zinc, was monitored by the quenching of Zinquin ethyl ester fluorescence. Cellular zinc uptake from ZnSO_4_ was significantly increased (by 36%), whereas zinc uptake from MZ was only partially increased (by 11%). The reason for this result may be that less free zinc within a certain time was dissociated by MZ than by ZnSO_4_. In addition, work by Tacnet demonstrated that organic zinc absorption is dependent on a variety of membrane transport mechanisms in pig small intestine [30], including mainly unsaturated diffusion and saturable carrier-mediated mechanisms. Thus, we speculated that the zinc absorption mechanisms of ZnSO_4_ and MZ were different.

### 2.9. The Effect of Phytic Acid on Zinc Bioavailability

Our daily staple foods, such as rice, corn, and cereals, generally contain phytic acid [31], and the phosphate groups of phytic acid can form insoluble complexes with zinc [32], which can significantly affect the absorption and utilization of zinc in the gastrointestinal tract [31]. Therefore, to assess the bioavailability of zinc from MZ, it is necessary to understand the absorption and utilization of zinc in the presence of phytic acid. As shown in Figure 7B, zinc uptake from ZnSO_4_ and MZ in the presence of a 2:1 molar ratio of zinc/phytic acid was monitored by the quenching of Zinquin ethyl ester fluorescence. The cellular uptake of zinc from ZnSO_4_ was barely increased, indicating that the absorption of zinc from ZnSO_4_ was almost completely inhibited by phytic acid. Moreover, the zinc uptake from MZ decreased from 11% (without phytic acid treatment) to 6%, indicating that phytic acid incompletely blocked the zinc uptake from MZ. In other words, MZ showed a certain anti-phytic acid complexation, which reduced the negative effect of phytic acid on zinc absorption. 

The free zinc contents of each group after phytic acid treatment are shown in Figure 7C. The free zinc content in the MZ group was significantly higher than that in the ZnSO_4_ group and in the mixed system of M and ZnSO_4_ (*p* < 0.05), indicating that the effect of the MZ was more significant against the anti-phytic acid complexation than that of the peptide M. This result is similar to the results of Hansen et al. [33], who reported that a certain amount of casein phosphopeptides (CPP) could overcome the inhibitory effect of phytate on zinc absorption. In addition, Zhu et al. also found that the chelation of zinc with peptides derived from wheat germ protein possessed higher zinc bioavailability than ZnSO_4_ in Caco-2 cells [34]. Finally, the results revealed that some zinc ions could be "protected" after the combination of peptide M and zinc to avoid the inhibition of zinc absorption by dietary interactions, thereby improving the effective bioavailability of zinc.

### 2.10. Effect of MZ on hZIP4 and ZnT1 mRNA Levels in Caco-2 Cells

**Figure 8:**
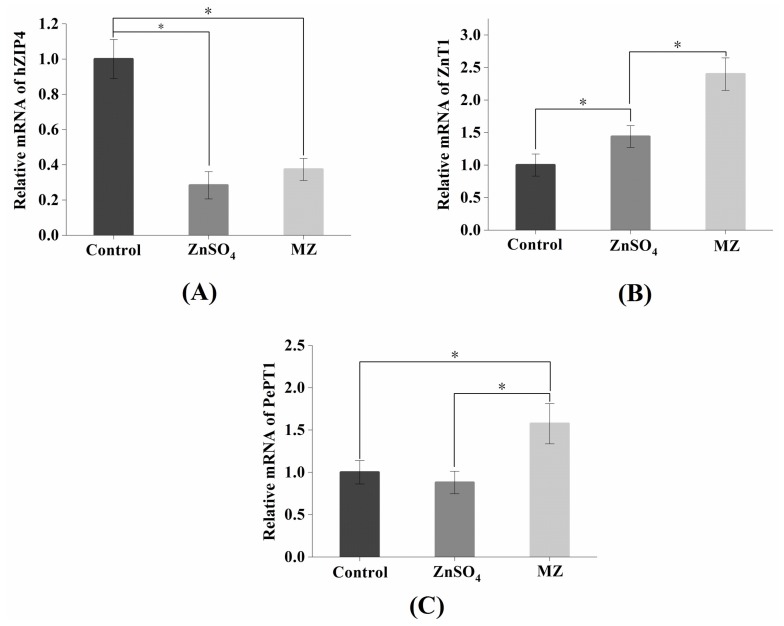
Effects of MZ on the relative level of hZIP4, ZnT1, and PepT1 mRNA in Caco-2 cells. MZ = peptide-zinc complex. Asterisk (*) indicates significant difference between groups (*p* < 0.05).

The zinc regulatory protein hZIP4 and the zinc transporter ZnT1 play an important role in maintaining cellular zinc homeostasis [35]. hZIP4 is a member of the ZIP family, mainly located in the apical membrane of small intestinal cells and is mainly used to regulate dietary zinc intake [36]. ZnT1 was the first zinc transporter found in the ZnT family and is mainly located on the basement membrane of intestinal epithelial cells, its main function is to transport zinc out of the cell [35]. As shown in Figure 8A, the hZIP4 level of both the ZnSO_4_ and MZ groups was significantly reduced compared with that of the control group, while there was no significant difference between the ZnSO_4_ and MZ groups. The expression of hZIP4 is regulated by intracellular zinc levels, and when the amount of exogenous zinc is sufficient, the cells will reduce the uptake of zinc, which may result in a decrease in hZIP4 expression [36]. Our results indicated that both ZnSO_4_ and MZ at concentrations equivalent to 96 μM zinc can meet the zinc demand of Caco-2 cells. In addition, the levels of ZnT1 were significantly upregulated in both the ZnSO_4_ and MZ groups compared with the control group, and the levels of ZnT1 were also significantly increased in the MZ group compared with the control group (*p* < 0.05) (Figure 8B), indicating that MZ is more fully absorbed than ZnSO4 by Caco-2 cells. Overall, it can be seen that cells will not only reduce the intake of zinc but also increase the export of zinc through the zinc ion channel pathway when there is a sufficient supply of exogenous zinc, representing a feedback regulation mechanism for the organism to adapt to changes in external zinc levels.

### 2.11. Effect of MZ on PePT1 mRNA Levels in Caco-2 Cells

PePT-1 is a member of the oligopeptide transporter family, and its major expression site is on the brush border membrane of intestinal epithelial cells [37]. Maubon et al. found that the expression of PePT1 in Caco-2 cells and small intestinal epithelial cells were quite similar [38]. As shown in Figure 8C, the levels of PePT1 were significantly increased in the MZ group compared with the control and ZnSO_4_ groups (*p* < 0.05), while there was no significant difference between the ZnSO_4_ and control groups, suggesting that MZ promoted the expression of PePT1 in Caco-2 cells. Tacnet et al. demonstrated that organic zinc can be absorbed either by the regulation of zinc transporters or by the absorption mechanisms of small peptides or amino acids [30]. Therefore, it is speculated that MZ may be absorbed and utilized by cells through the small peptide transport system.

**Figure 9:**
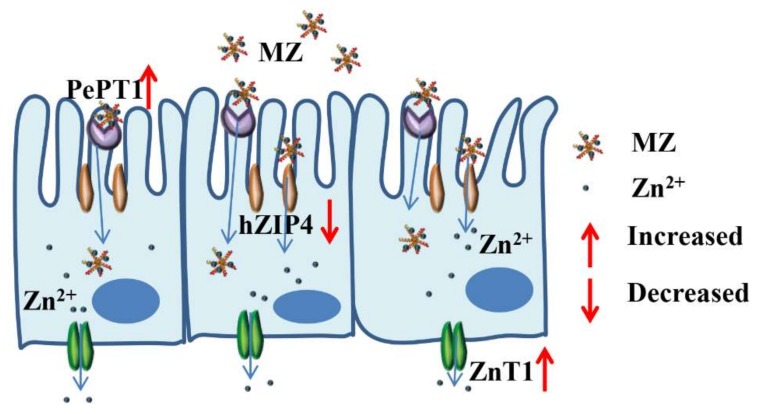
Possible mechanisms of MZ promotes zinc absorption of Caco-2 cells.

Based on the above results, we can conclude that the intestinal absorption of MZ may occur mainly through two pathways: the zinc ion channel and the small peptide transport pathway. For the zinc ion channel pathway, MZ promotes the activation of ZnT1 and inhibits the activation of hZIP4, which consequently maintains cellular zinc homeostasis. For the small peptide transport pathway, MZ promotes the activation of PePT1, leading to the promotion of zinc absorption from MZ. The two pathways finally result in the promotion of zinc absorption from MZ (Figure 9).

## 3. Materials and Methods

### 3.1. Materials and Chemicals

Oyster (*Crassostrea gigas*) was obtained from a local market (Qingdao, China). *N,N,N’,N’*-tetrakis (2-pyridylmethyl)-ethylene-diamine (TPEN) and methyl-thiazolyl-diphenyl-tetrazolium bromide (MTT) were obtained from Sigma Chemical Ltd. (St. Louis, MO, USA). Zinquin ethyl ester was purchased from AAT Bioquest Ltd. (Sunnyvale, CA, USA). Dulbecco’s modified Eagle’s medium (DMEM), *N*-2-hydroxyethylpiperazine-*N*-ethane-sulfonicacid (HEPES), Foetal bovine serum (FBS), TrypLE TM Express cell dissociation reagent, Hank’s balanced salt solution (HBSS), and Dulbecco’s phosphate-buffered saline (DPBS) were procured from Thermo Fisher Ltd. (Waltham, MA, USA). All other reagents used in this study were of analytical grade.

Caco-2 cells preserved in our laboratory [26] were grown in 4.5 g/L of DMEM supplemented with 4 mM glutamine, 25 mM HEPES, 1 mM sodium pyruvate, 10% FBS, and 1% penicillin-streptomycin mixture at 37 °C in a constant temperature incubator containing 5% CO_2_. 

### 3.2. Oyster Hydrolysate Preparation

The oyster hydrolysates were prepared in accordance with the method of Chen et al. [14]. Briefly, oyster meat was first homogenized and boiled for 10 min. After cooling, the pH was adjusted to 2.0 with 1 mol/L HCl, pepsin (2500 U/g protein) was added, and the homogenate was digested in a 37 °C water bath for 2.5 h. Afterwards, the pepsin was inactivated at 100 °C for 10 min. Then, the suspension was centrifuged at 6000 r/min for 15 min, after which the supernatant was passed through a 0.45 μm microporous membrane and then desalted by a Chelax-100 exchange resin chromatography column. The obtained hydrolysate products were freeze-dried and stored for further use.

### 3.3. Modification of Oyster Hydrolysates by Plastein Reaction

The plastein reaction conditions were as follows: exogenous l-[1-^13^C]glutamate concentration of 2–18 mg/mL; substrate (hydrolysates products) concentration of 25%–45% (w/v); temperature of 20–60 °C; pepsin dosage of 100–900 U/g protein; pH of 2–9; and reaction time of 1–6 h. The free amino acid reduction during the plastein reaction was used as an indicator to optimize the plastein reaction conditions. Afterwards, the plastein products were boiled at 90 °C for 15 min to inactivate the pepsin. Then, the plastein products were further eluted by a Sephadex G-15 gel column (2.6 cm × 65 cm). The absorbance was measured at 220 nm using a UV-2550 spectrophotometer (Shimadzu, Japan), and the components were collected before the peak of 50 mg/mL phenylalanine (as a standard). The obtained components were freeze-dried and stored for further use.

### 3.4. Free Amino Acids Determination

The free amino acids were detected based on the *O*-phthaldialdehyde (OPA) method [19]. Briefly, 100 μL of oyster plastein products was added to 2 mL of OPA reagent (40 mg/mL). After 2 min of reaction, the absorbance of the resultant solution was measured at 340 nm. Glutamate was used as the standard, and the standard curve was calculated as follows:

*Y* = 0.186*X* − 0.005 (*R*^2^ = 0.997)

*X* indicates the concentration of free amino acids, and *Y* indicates the absorption value.

### 3.5. Zinc-Binding Capacity Determination

Ten milligrams of prepared products were dissolved in 5 mL of water, followed by the addition of 5 mL of ammonium chloride buffer, pH 10.0. Then, the chrome black T indicator was added, and ethylenediaminetetraacetate (EDTANa_2_) was used as a titration solution to determine blue coloration. Afterwards, the consumed EDTANa_2_ volume was recorded. In addition, the soluble protein content of the plastein products was detected by the Folin-phenol method [39]. The calculation formula was as follows [40]:

zinc-binding capacity (%) = *mV*/*W*

where, *m* (mg/mL) is the zinc mass corresponding to 1 mL of consumed EDTANa_2_ (0.05 mol/L), *V* (mL) is the volume of consumed EDTANa_2_, and *W* (g) is the soluble protein content of the plastein products.

### 3.6. Hydrophobic Changes during the Plastein Reaction

The hydrophobic changes were determined by the ANS fluorescence probe method [41]. The plastein products were first dissolved in 0.01 M sodium phosphate buffer to obtain the different concentrations of protein and subsequently, 10 μL ANS was added. The fluorescence intensity was measured at an excitation wavelength of 375 nm and an emission wavelength of 440 nm. The hydrophobicity index was determined by the initial slope of the protein concentration versus fluorescence intensity.

### 3.7. Effects of Protein Denaturants on Plastein Products

Five hundred milligrams of hydrolysis products and plastein products were dissolved separately in 35 mL ddH_2_O, 10 mM sodium phosphate buffer, pH 6.0, 10% (*v/v*) trichloroacetate (TCA), 50% (*v/v*) acetic acid, and 0.3 M sodium lauryl sulfate (SDS). After a 30 min reaction, the solutions were centrifuged to obtain the supernatant. Afterwards, the protein contents of the supernatant, hydrolysis products and plastein products were determined by the Kjeldahl method according to Hartnett et al. [42]. Protein solubility was expressed as the protein content of the supernatant versus the original products. In addition, the effect of urea on the plastein products was determined as follows: the hydrolysis and plastein products were dissolved in different concentrations of urea (0, 1, 1.5, 2, 2.5, 3, and 4 mol/L), and the corresponding absorbance was measured at 420 nm by a TU-1810 spectrophotometer (Beijing, China).

### 3.8. Change in the Molecular Weight Distribution Profile

The molecular weight distribution of plastein products was determined by a TSK-Gel 2000 SWXL column (7.8 × 300 mm, Agilent Technologies, Santa Clara, CA, USA) equipped with an ultraviolet detector (LC-20AT, Shimadzu Corp., Tokyo, Japan) system at a flow rate of 0.5 mL/min for 30 min. Acetonitrile, ddH_2_O and trifluoroacetic acid (*v/v/v*: 45/55/0.1) were used as mobile phases, and the column temperature was 30 °C. Tyrosine (182 Da), glutathione (307 Da), bacitracin (1423 Da), insulin (5733 Da), and cytochrome C (12590 Da) were used as standards. The equation of the standard curve was as follows: Log (Mw) = −0.2249*t* + 6.6846 (*R*^2^ = 0.9707), where Mw is the molecular weight, and *t* is the retention time.

### 3.9. Determination of the Abundance of l-[1-^13^C]Glutamate

The samples were wrapped in foil and then placed on a burner (1150 °C) of the elemental analyser set to full combustion. Afterwards, the produced gases were placed in a reduction furnace (810 °C) to remove excess oxygen and reduce the nitrogen oxides to N_2_. Then, a gas chromatographic column was used to purify the gas, and the samples were subsequently placed in the isotope mass spectrometer to determine the l-[1-^13^C]glutamate abundance of the samples.

### 3.10. Characterization of the MZ

#### 3.10.1. Preparation of the MZ

The oyster peptide Glu-Val-Pro-Pro-Glu-Glu-His (M) that was isolated and purified in our previous study [16] was dissolved in aqueous ZnSO_4_ solution, and the mass ratio of the peptide and ZnSO_4_ was 3:1. Then, the mixture was reacted at 40 °C for 30 min. The solution was filtered by 0.22 μm Millipore filters. Afterwards, anhydrous ethanol was added and centrifuged at 8000 r/min for 10 min to obtain the complex. The MZ was freeze-dried and stored for further use.

#### 3.10.2. UV-Visible

The zinc-binding peptide and MZ were dissolved in ddH_2_O and filtered through 0.22 μm Millipore filters. The absorption spectra were recorded in the 200–400 nm region with a UV-2550 spectrophotometer (Shimadzu, Japan). ddH_2_O was used as a reference.

#### 3.10.3. Morphology Analysis

The morphology of the MZ was observed by scanning electronic microscopy (SEM). The lyophilized MZ powder was spread on a glass slide and sputter-coated with gold (JEOL JFC-1200 fine coater, Tokyo, Japan). The SEM was used to observe the film at 15 kV.

### 3.11. Cytotoxicity of the MZ against Caco-2 Cells

The procedure to construct an in vitro intestinal cell model was performed according to the method described by Zou et al. [43], with some modifications. Briefly, after the fusion degree of Caco-2 cells reached approximately 80%, the growth medium was removed, and the cell monolayers were washed twice with DPBS. Then, the TrypLE TM Express cell dissociation reagent was added to each well. After 7 min of digestion, high-glucose DMEM medium was proportionally gently added with liquid transfer. The suspension was transferred into a 15 mL sterile centrifuge tube for centrifuging, and the supernatant was discarded. The pellet was diluted with high-glucose DMEM medium to prepare a single-cell suspension. Cells were seeded at a density of 1 × 10^5^ cells/well in collagen-coated 24-well plates (BD Biosciences, San Joes, CA, USA). After reaching confluence, the cells were allowed to differentiate in high-glucose DMEM medium for another 10 d, and an in vitro intestinal cell model was obtained. 

Cell viability was measured according to the method described by Zou et al. with some modifications [43]. Cells were seeded at a density of 1 × 10^4^ cells/well in collagen-coated 96-well plates incubated for 24 h in a thermostat incubator with a humid atmosphere containing 5% CO_2_ at 37 °C. After the medium was removed, 200 μL of the samples at different concentrations were added to each well, and 8–10 parallel wells were prepared for each sample. After 20 h of incubation in a CO_2_ incubator at 37 °C, 20 μL of MTT (5 mg/mL) was added to each well. The precipitate was removed by centrifugation after 4 h of incubation. Then, 150 μL of DMSO was added to each well, and the cell culture plate was shaken at low speed for 10 min. The absorbance was measured with a microplate reader at 570 nm. The relative viability of cells (IC) was calculated according to the method of Duan et al. [44]. IC was calculated as follows: IC (%) = (OD sample group − OD blank)/(OD control group − OD blank) × 100%.

### 3.12. Zinc Absorption Assay in Caco-2 Cells

The zinc absorption experiment was based on the method of Wu et al. and Makhov et al., with some modifications [45,46]. The caco-2 cell medium was first removed, and 10 μM of TPEN was added for a 2 h treatment to consume intracellular zinc reserves after the cells were washed with HBSS 3 times. Then, the supernatant was discarded, the cell sedimentation was washed with HBSS 3 times again, 500 μL of HBSS solution containing 20 μM Zinquin ethyl ester was added, and the samples were incubated for 30 min in a CO_2_ thermostat incubator at 37 °C. The supernatant was then discarded, the sample was dissolved in HBSS, and 1 mL of oyster source MZ or ZnSO_4_ solution was added (the different sample solutions ensured a zinc content of 96 μM). The cell culture plate was immediately placed in a fluorescence microplate reader with Zinquin ethyl ester fluorescence detected every 3 min (360 nm excitation, 480 nm emission).

The effect of phytic acid on zinc absorption was performed according to the method of Sreenivasulu et al., with some modifications [47]. Phytic acid was dissolved in HBSS to prepare a 96 μM stock solution. The phytic acid reserve solution was diluted to 48 μM in MZ and ZnSO_4_ solutions containing 96 μM Zn^2+^, such that the molar ratio of Zn^2+^ to phytic acid was 2:1. The mixed samples were placed in a 37 °C incubator and incubated for 1 h. Then, 1 mL of the mixture was added to the differentiated Caco-2 cells. The cell culture plate was immediately placed in a fluorescence microplate reader with Zinquin ethyl ester fluorescence detected every 3 min (360 nm excitation, 480 nm emission). In addition, in vitro experiment, the MZ and ZnSO_4_ were dissolved in ddH_2_O, respectively, then the phytic acid was added to reach a 2:1 molar ratio of Zn^2+^ to phytic acid. The mixed samples were placed in a 37 °C incubator and incubated for 30 min. Then, the precipitate was removed by centrifugation. The zinc content in the supernatant was determined by an atomic absorption spectrophotometer.

### 3.13. Effect of MZ on hZIP4, PepT-1 and ZnT1 mRNA Levels in Caco-2 Cells

The expression of mRNA was detected according to the methods of Li et al. [48]. Total RNA was extracted as previously described [49]. The primers were designed using Premier 5.0 software (Premier Biosoft International, Palo Alto, CA, USA) (Table 2). RT-qPCR amplifications were performed with 3 biological replicates using a Bio-Rad CFX Connect System (BioRad Laboratories, Inc., San Diego, CA, USA). The housekeeping gene β-actin was chosen to normalize RNA amounts (internal control). The relative expression of the gene = 2^−∆∆CT^; ∆CT = CT of the target gene―CT of β-actin; and ∆∆CT = ∆CT of the observed sample―CT of the control sample.

### 3.14. Statistical Analysis

All experiments were carried out in triplicate, and data were expressed as the mean ± standard deviation (SD), unless specifically noted. The least significant difference method (LSD) was used to compare and analyze the difference in mean values with SPSS 20.0 statistical software (SPSS Inc., Chicago, IL, USA) and the significance level was 5%.

## 4. Conclusions

In this study, the efficient zinc-binding peptides were prepared from oyster-modified hydrolysates by adding exogenous glutamate according to the plastein reaction. The addition of exogenous l-[1-^13^C]glutamate contributed to an increase in the zinc-binding capacity of the plastein products and that a hydrophobic interaction was the main mechanism of action of the plastein reaction. Structural analyses suggested that zinc-binding peptides could bind with zinc and form the peptide-zinc complex (MZ). Furthermore, the MZ absorption effect and absorption pathway under simulated intestinal epithelial cells were studied. The results showed that the absorption effect of MZ under phytic acid treatment was significantly higher than that of ZnSO_4_, indicating that the MZ could significantly enhance zinc bioavailability than ZnSO_4_. Additionally, MZ could regulate the gene expression of hZIP4, ZnT1, and PepT1, indicating that MZ could promote the zinc absorption through the traditional zinc ion channel and small peptide transport pathway. In summary, the plastein reaction could significantly increase the ability of peptides to bind zinc, and the MZ has better absorption and bioavailability than ZnSO_4_. The MZ exhibits great potential as a functional ingredient in food and nutraceuticals.

## Figures and Tables

**Table 1:** Zinc-binding capacity and l-[1-^13^C]glutamate abundance of different components of plastein products. The control group is the plastein products without exogenous l-[1-^13^C]glutamate added. The different letters indicate significant differences (*p* < 0.05).

Sample Name	Zinc-Binding Capacity (mg/g)	l-[1-^13^C]glutamate Abundance (‰)
Control	69.12 ± 1.54 ^c^	−21.87
a	94.43 ± 2.07 ^b^	255.73
b	101.08 ± 3.10 ^a^	1108.22
c	72.21 ± 2.68 ^c^	7.16
d	26.54 ± 1.43 ^d^	−4.54

**Table 2:** Primers used in this study.

Genes	Oligonucleotide Sequence (5′–3′)
β-actin	Forward GGAGATTACTGCCCTGGCTCCTA
	Reverse GACTCATCGTACTCCTGCTTGCTG
ZnT1	Forward ATGGGGGCTCTGGTGAACGC
	Reverse CCTGGTCGGGACCCTGCTCG
PepT1	Forward GCTCTTATCGCCGACTCGTG
	Reverse GGGTTTGATTCCTCCAGTCC
hZIP4	Forward TGGTCTCTACGTGGCACTC
	Reverse GGGTCCCGTACTTTCAACATC
